# Supplementary material for: The influence of the COVID-19 pandemic on the short- and long-term interactions in the agricultural market: Evidence from a connectedness network approach
Source: PLoS One. 2023 Dec 6;18(12):e0295294. doi: 10.1371/journal.pone.0295294 (PMC10699634; doi:10.1371/journal.pone.0295294)
Supplement: Attachment — Submitted filename: Response to Reviewers.docx [file pone.0295294.s002.docx]

**Journal Requirements:**

When submitting your revision, we need you to address these additional requirements.

**1.** Please ensure that your manuscript meets PLOS ONE's style requirements, including those for file naming. The PLOS ONE style templates can be found at
<https://journals.plos.org/plosone/s/file?id=wjVg/PLOSOne_formatting_sample_main_body.pdf> and
<https://journals.plos.org/plosone/s/file?id=ba62/PLOSOne_formatting_sample_title_authors_affiliations.pdf>

**Response**: Thanks for the editor’s suggestions. In this version of the manuscript, we format the manuscript body according to the file of ‘**PLOS One formatting sample main body**’. Moreover, we prepare a title page according to the file of ‘**PLOS One formatting sample title authors affiliation**’.

**2.** Please ensure that you include a title page within your main document. We do appreciate that you have a title page document uploaded as a separate file, however, as per our author guidelines (<http://journals.plos.org/plosone/s/submission-guidelines#loc-title-page>) we do require this to be part of the manuscript file itself and not uploaded separately.
Could you therefore please include the title page into the beginning of your manuscript file itself, listing all authors and affiliations.

**Response**: Thanks for the editor’s suggestions. In this version of the manuscript, we prepare a title page according to the file of ‘**PLOS One formatting sample title authors affiliation**’. Moreover, this title page, listing all authors and affiliations, is placed at the beginning of this manuscript file.

**3.** We note that you have indicated that data from this study are available upon request. PLOS only allows data to be available upon request if there are legal or ethical restrictions on sharing data publicly. For more information on unacceptable data access restrictions, please see <http://journals.plos.org/plosone/s/data-availability#loc-unacceptable-data-access-restrictions.>

In your revised **cover letter,** please address the following prompts:

**a)** If there are ethical or legal restrictions on sharing a de-identified data set, please explain them in detail (e.g., data contain potentially sensitive information, data are owned by a third-party organization, etc.) and who has imposed them (e.g., an ethics committee). Please also provide contact information for a data access committee, ethics committee, or other institutional body to which data requests may be sent.

**Response**: Thanks for the editor’s suggestions. **There are no ethical or legal restrictions on sharing a de-identified data set.** The above information is declared in the cover letter of this manuscript.

**b)** If there are no restrictions, please upload the minimal anonymized data set necessary to replicate your study findings as either **Supporting Information files** or to a stable, public repository and provide us with the relevant URLs, DOIs, or accession numbers. For a list of acceptable repositories, please see <http://journals.plos.org/plosone/s/data-availability#loc-recommended-repositories.>

**Response**: Thanks for the editor’s suggestions. **We upload the minimal anonymized data set necessary to replicate our study findings as Supporting Information files**.

We will update your Data Availability statement on your behalf to reflect the information you provide.

**Additional Editor Comments:**

To enhance the literature review, we suggest that you consider incorporating the following papers from the publication list:

1.Bao, Z., & Huang, D. (2021). Shadow banking in a crisis: Evidence from FinTech during COVID-19. Journal of Financial and Quantitative Analysis, 56(7), 2320–2355. <https://doi.org/10.1017/S0022109021000430>

2. Huang, D. (2020). How effective is social distancing. Covid Economics, Vetted and Real-Time Papers (59), 118–148. [https://doi.org/](https://doi.org/10.1017/S0022109021000430)[[10.2139/ssrn.3680321](https://doi.org/10.1017/S0022109021000430)](https://doi.org/10.2139/ssrn.3680321)

3. Li, N., Chen, M., Gao, H., Huang, D., & Yang, X. (2023). Impact of lockdown and government subsidies on rural households at early COVID-19 pandemic in China. China Agricultural Economic Review, 15(1), 109–133. [https://doi.org/10.1108/CAER-12-2021-0239](https://doi.org/10.1016/j.eneco.2020.104741)

These papers are relevant to your study as they provide insights into the effects of the COVID-19 pandemic on various aspects of the economy, including the financial sector and rural households.

Bao and Huang (2021) investigate the role of FinTech during the COVID-19 crisis, which could provide valuable context for understanding the broader economic implications of the pandemic.

Huang (2020) examines the effectiveness of social distancing measures, which may have indirect effects on the agricultural market through changes in consumer behavior and supply chain disruptions.

Li et al. (2023) explore the impact of lockdown measures and government subsidies on rural households in China, which could be particularly relevant to your study as it focuses on the agricultural market.

Incorporating these papers into your literature review will help to strengthen the theoretical foundation of your study and provide a more comprehensive understanding of the COVID-19 pandemic's effects on the agricultural market.

**Response**: Thanks for the editor’s suggestions. In this version of the manuscript, we review these three articles as follows.

Bao and Huang (2021) used the borrower-by-month panel data from the credit loan records of 3 large Chinese fintech companies and a leading commercial bank in China and applied the difference-in-difference approach to compare the differentials in the changes of credit access and delinquency rates between the fintech and bank borrowers before and after the COVID-19 pandemic. The obtained results are used to examine the fintech loan outcomes before and after the COVID-19 pandemic and further to evaluate the performance of shadow banking in a crisis via 2 aspects: the quantity of loans and the quality of loans. They got the following findings. First, fintech companies are more likely to expand credit access to new and financially constrained borrowers after the start of the pandemic. Or, the demand for fintech and traditional bank loans increased at the aggregate and individual levels after the pandemic, especially for fintech loans. Second, the delinquency rate for fintech companies increased significantly after the outbreak while banks were unaffected. This indicates that the quality of fintech loans is more susceptible to adverse shocks. Third, borrowers are more likely to default on their fintech loans rather than their bank loans.

Huang (2020) used a panel of daily COVID-19 infection cases of all counties in the United States as the data, then applied the factor model with interactive fixed effects, that measures the relative frequency and geographic distribution of COVID-19 infections and provided hypothetical infection distribution in the counties, to examine the effects of social distancing policy on reducing the transmission of the COVID-19 spread. He found social distancing lowered the average daily infection cases by 12%. Moreover, the effects are heterogeneous in an individual's income, race, education, and political beliefs.

Li et al. (2023) used the data from a national telephone survey of rural households in the early stages of the COVID-19 pandemic, then applied the ordered probit model and linear regression model to examine the impact of lockdown and government subsidies on rural households in early COVID-19 pandemic in China. They found that logistics disruption due to the lockdown negatively affected rural households. Moreover, breeding, transport, and sales suffer more from logistics than cultivation. The reason is that the traffic restriction between and within cities is directly reflected in the serious hindrance of logistics, which leads to production problems such as price rise, production means shortage, sales reduction, and, finally, loss of rural households.

In this revised manuscript, we concisely cite the above three articles in the section **Introduction** of this study and it is illustrated as follows.

Everybody knows that food is a crucial article to survive for human beings. However, climate change such as sudden floods or droughts has made agricultural commodities like grains be planted not smoothly in recent years. Moreover, in 2020, the farmers and workers were infected by the coronavirus disease 2019 (COVID-19) pandemic. To reduce the spread of the virus, the government in many countries enacted a series of restrictive policies such as the social distancing policy and lockdown measures because social distancing lowered the average daily infection cases by 12% in the United States (Huang, 2020). These restrictive policies altered the mode of management in financial institutions. For example, the demand for fintech and traditional bank loans increased at the aggregate and individual levels after the pandemic, especially for fintech loans (Bao and Huang, 2021). Moreover, the above policies also made agricultural products from planting to transportation run not swimmingly (Mahajan and Tomar, 2020; Ruan et al. 2021). For instance, the lockdown measures resulted in logistics disruption because of the interruption to transportation, and this disruption further caused a series of problems in agricultural production such as price rise, production means shortage, sales reduction, and, finally, rural households’ losses (Li et al., 2023). These phenomena have resulted in worrying about food shortages for people and further prompted the food crisis issues to be paid attention to worldwide (Dahl et al., 2020; Mensi et al., 2014, 2021).

In addition to improving the literature review, we have the following detailed comments for you to address in your revision:

1. Please provide a more **detailed explanation of the bivariate GARCH model and its relevance to your study**. Explain **why this model is appropriate for analyzing the connectedness network** in the agricultural market during the COVID-19 pandemic.

**Response**: Thanks for the editor’s suggestions. In this revised manuscript, we explain why the bivariate GARCH model is appropriate for analyzing the connectedness network in the agricultural market during the COVID-19 pandemic in the section **Introduction** of this study, and it is illustrated as follows.

This study mainly uses the connectedness networks in the agricultural market to describe the interactions between **alternative two assets** within a group of commodities in the agricultural market from the view of the significance of the interaction. Moreover, the return series of most financial assets displays linear dependence and a strong autoregressive conditional heteroskedasticity (**ARCH**) effect (Su, 2014a, b, c; Su, 2015). The above facts indicate that the **bivariate GARCH** model is suitable because this model can seize the volatility heterogeneity or volatility changing over time in the financial return series. In addition, this study aims to explore the influence of the COVID-19 pandemic on short- and long-term interactions. Hence, we add **two time-dummy variables** to the above model to explore the interaction between two commodities **before and after the epidemic**, so the bivariate GARCH model with two time-dummy variables is very suitable for this research topic.

***Note*:** The above discussion is listed in the section of the **Introduction** in this revised manuscript.

2. Clarify the **methodology** used to construct the **connectedness network** and provide more information on the **data sources** and **sample period**.

**Response**: Thanks for the editor’s suggestions. In this revised manuscript, we create a new **subsection 2.2** in the section **Methodology** of this study to explain the methodology used to construct the connectedness network and it is illustrated as follows.

*2.2 The theory of constructing the connectedness networks*

This study aims to explore the influence of the COVID-19 pandemic on short- and long-term interactions via a connectedness network approach. Additionally, the short-term interactions are the volatility and return spillovers, which are directional financial features. Conversely, the long-term interaction is the correlation, which belongs to the non-directional financial feature. Hence, in this subsection, we illustrate how to use the significance of parameters related to two short-term interactions ($\phi_{\mathrm{ij}}$ and $\nu_{\mathrm{ij}}$) and one long-term interaction ($\omega_{\mathrm{ij}}$) to get the corresponding *connectedness* networks.

If parameter $\phi_{\mathrm{ij}}$ (or $\nu_{\mathrm{ij}}$) is significantly positive (negative) then a positive (negative) return (or volatility) spillover from the $j^{\mathrm{th}}$ asset to the $i^{\mathrm{th}}$ asset subsists in a pair of assets. Subsequently, in a network of return (or volatility) spillover, we utilize a red (blue) arrow from a node denoting the $j^{\mathrm{th}}$ asset to another node denoting the $i^{\mathrm{th}}$asset to represent this positive (negative) return (or volatility) spillover. Regarding each node in a return (or volatility) spillover network, we count the total number of arrows away from that node and point to that node as well as the summation and difference for the above two total numbers. The results of four types of calculations are recorded as four numbers in a bracket beside this node. That is, the **first (second)** number in this bracket records the total number of arrows away from (point to) that node. The **third** number in this bracket denotes the summation between the first and second numbers in the same bracket or records the summation for the above two total numbers. If this number in a specific node is the greatest (smallest) number among the third numbers for all nodes, then the asset corresponding to this specific node is the most (least) active asset in this network, regulating that the outer circle of this node is marked in solid (dash) line. The **fourth** number in this bracket denotes the difference between the first and second numbers in the same bracket or records the difference for the above two total numbers. If the difference is greater (less) than zero, then the asset corresponding to this node is a net transmitter (receiver) in a system and this node is marked in red (yellow). In addition, if the difference is equal to zero, then the asset corresponding to this node is neutral in a system and this node is marked in green.

If parameter $\omega_{\mathrm{ij}}$ is significantly positive (negative) then a positive (negative) correlation between the $i^{\mathrm{th}}$ asset and the $j^{\mathrm{th}}$ asset exists in a pair of assets. Subsequently, in a network of correlation, we utilize a red (blue) line between a node denoting the $i^{\mathrm{th}}$ asset and another node denoting the $j^{\mathrm{th}}$ asset to represent this positive (negative) correlation. Regarding each node in a correlation network, we calculate the total number of lines connected with this node and record this number in a bracket beside this node. If this number in a specific node is the greatest (smallest) number among these numbers for all nodes, then the asset corresponding to this node is the most (least) active asset in this network, regulating that the outer circle of this node is marked in solid (dash) line.

In the first paragraph of **Data and Descriptive Statistics** of section 3 in this revised manuscript, we provide more information on the **data sources** and **sample period,** and it is illustrated as follows.

This work primarily employs a connectedness network method to inspect the influence of the COVID-19 pandemic on the interactions among the seven agricultural commodities. Hence, the study data contain daily close price data of wheat, corn, oat, soybean, soybean oil, coffee, and sugar in the agricultural market. The prices of wheat, corn, Oats, and soybean are measured in U.S. Dollars per bushel whereas those of soybean oil, coffee, and sugar are U.S. Dollars per pound. All data was downloaded from the website <https://www.macrotrends.net> and they cover the period from March 26, 2013, to March 11, 2021. Consequently, according to the date the COVID-19 pandemic occurred on March 11, 2020, we partitioned the study data into the pre-pandemic and post-pandemic periods. Notably, Wheat, corn, oat, soybean, and soybean oil belong to the grains type of agricultural commodities. On the contrary, coffee and sugar are included in the beverage type of agricultural commodities. In addition, soybean oil is the raw material of renewable diesel and it is extracted from the soybean.

3. Discuss the **potential limitations** of your study and **any assumptions** made in your analysis. Address how **these limitations** may affect the interpretation of your results and the generalizability of your findings.

**Response**: Thanks for the editor’s suggestions. In the **Conclusion and further discussion** of section 5 in this revised manuscript, we illustrate the **assumptions** and the **potential limitations** of this study and how **these limitations** may affect the interpretation of results. They are illustrated as follows.

Overall, the above impact results owing to this pandemic are obtained from the **assumption** of the variation of the **average phenomena** of the interactions in the pre-pandemic and post-pandemic periods. Therefore, the method in this study **cannot observe how the impact of this pandemic** on the interactions **varies with time**. However, we can use the rolling window method with a given fixed sample window size to predict the parameters related to the interactions such as $\phi_{12}$, $\phi_{21}$, $\nu_{12}$, $\nu_{21}$, and $\omega_{12}$. From the trend of the values of the parameter estimate with time, we can observe **how the impact of this pandemic** on the interactions **varies with time**. This question can be reserved as the topic in my future research.

4. Expand on the **policy implications** of your findings, particularly in the context of **short- and long-term investment strategies** in the agricultural market. Provide **specific recommendations for policymakers based on your results.**

**Response**: Thanks for the editor’s suggestions. In the **Conclusion and further discussion** of section 5 in this revised manuscript, **based on the results listed in** Table 2 and Table 3, we provide **specific recommendations for policymakers** to make short-term investment strategies to earn more profit and reduce the risk, respectively, and it is illustrated as follows. In addition, **based on the results listed in** Table 4, we make a long-term investment strategy to reduce the portfolio risk and it is illustrated as follows.

**Based on the results listed in** Table 2, we make short-term investment strategies to earn more profit and it is illustrated as follows.

Firstly, in the pre-pandemic period, they can use the return of coffee to predict the return of corn, oats, and soybean oil. Conversely, in the post-pandemic period, they can use the return of soybean oil to predict the return of wheat and oats. The reasons are that, in Figure 3 (a), there exist three positive return spillovers from coffee to corn, oats, and soybean oil but, in Figure 3 (b) there exist two negative return spillovers from soybean oil to wheat and oats. Moreover, as reported in the data in Table 2, we further presume that, in the pre-pandemic period, a 100% increase in coffee return today causes a 2.93%, 3.84%, and 2.42% rise in return for the corn, oats, and soybean oil tomorrow, respectively. Conversely, in the post-pandemic period, a 100% increase in soybean oil return today causes a 13.4% and 19.27% drop in return for the wheat and oats tomorrow, respectively. This indicates that we can make the following short-term investment strategies to earn more profit. During the pre-pandemic period, if the price of coffee rises today then long traders can buy and hold the position of the corn, oats, or soybean oil today until tomorrow. Conversely, during the post-pandemic period, if the price of soybean oil rises today then short traders can sell the position of the wheat or oats today until tomorrow.

**Note:** The **policy implications** proposed in the previous version of the manuscript are based on the following results listed in Table 2.

| The pre-pandemic period | | |  | The post-pandemic period | | |
| --- | --- | --- | --- | --- | --- | --- |
|  | cr-cf | oa-cf | so-cf |  | wh-so | oa-so |
| $\phi_{12}^{B}$ | 0.0293  (0.014)^b^ | **0.0384**  (0.022)^a^ | 0.0242  (0.012)^a^ | $\phi_{12}^{A}$ | **-0.1340**  (0.072)^a^ | **-0.1927**  (0.066)^c^ |
| $\phi_{21}^{B}$ | -0.0098  (0.029) | 0.0087  (0.024) | 0.0715  (0.037)^a^ | $\phi_{21}^{A}$ | **0.0219**  (0.053) | **0.0050**  (0.049) |
| pre | $\overset{\leftarrow}{\boldsymbol{(+)}}$ | $\overset{\leftarrow}{\boldsymbol{(+)}}$ | $\overset{\leftrightarrow}{\boldsymbol{(+)}}$ | post | $\overset{\leftarrow}{\boldsymbol{(-)}}$ | $\overset{\leftarrow}{\boldsymbol{(-)}}$ |

**Based on the results listed in** Table 3, we make short-term investment strategies to reduce the risk and it is illustrated as follows.

Secondly, in the pre-pandemic period, they can employ the volatility of oats to forecast the volatility of wheat, soybean, and coffee. Conversely, in the post-pandemic period, the volatility of oats is used to predict the volatility of wheat, corn, soybean, soybean oil, and coffee. The reasons are that, in Figure 4 (a), there exist three positive volatility spillovers from oat to wheat, soybean, and coffee but, in Figure 4 (b) there exist five positive volatility spillovers from oat to wheat, corn, soybean, soybean oil, and coffee. Moreover, as reported in the data in Table 3, we further presume that, in the pre-pandemic period, a 100% decrease in oats volatility today causes a 1.93%, 2.01%, and 5.86% drop in volatility for the wheat, soybean, and coffee tomorrow, respectively. Conversely, in the post-pandemic period, a 100% decrease in oats volatility today causes a 3.49%, 2.64%, 3.05%, 0.74%, and 7.90% drop in volatility for the wheat, corn, soybean, soybean oil, and coffee tomorrow, respectively. This indicates that we can make the following short-term investment strategies to reduce the risk. During the pre-pandemic period, if the volatility of oats decreases today then long traders can buy and hold the position of the wheat, soybean, or coffee today until tomorrow. Conversely, during the post-pandemic period, if the volatility of oats decreases today then long traders can buy and hold the position of the wheat, corn, soybean, soybean oil, or coffee today until tomorrow.

**Note:** The **policy implications** proposed in the previous version of the manuscript are based on the following results listed in Table 3.

| The pre-pandemic period | | | |  | The post-pandemic period | | | | |
| --- | --- | --- | --- | --- | --- | --- | --- | --- | --- |
|  | wh-oa | oa-sb | oa-cf |  | wh-oa | cr-oa | oa-sb | oa-so | oa-cf |
| $\nu_{12}^{B}$ | 0.0193  (0.006)^c^ | 0.0173  (0.028) | **0.0869**  (0.028)^c^ | $\nu_{12}^{A}$ | **0.0349**  (0.013)^b^ | **0.0264**  (0.014)^a^ | **-0.0527**  (0.021)^b^ | **-0.1336**  (0.080)^a^ | 0.0120  (0.030) |
| $\nu_{21}^{B}$ | -0.0188  (0.031) | 0.0201  (0.008)^b^ | 0.0586  (0.031)^a^ | $\nu_{21}^{A}$ | **-0.0700**  (0.034)^b^ | **-0.0663**  (0.025)^c^ | **0.0305**  (0.011)^c^ | **0.0074**  (0.004)^a^ | **0.0790**  (0.041)^a^ |
| pre | $\overset{\leftarrow}{\boldsymbol{(+)}}$ | $\overset{\to}{\boldsymbol{(+)}}$ | $\overset{\leftrightarrow}{\boldsymbol{(+)}}$ | post | $\overset{\leftarrow}{\boldsymbol{(+)}}\overset{\to}{\boldsymbol{(-)}}$ | $\overset{\leftarrow}{\boldsymbol{(+)}}\overset{\to}{\boldsymbol{(-)}}$ | $\overset{\leftarrow}{\boldsymbol{(-)}}\overset{\to}{\boldsymbol{(+)}}$ | $\overset{\leftarrow}{\boldsymbol{(-)}}\overset{\to}{\boldsymbol{(+)}}$ | $\overset{\to}{\boldsymbol{(+)}}$ |

**Based on the results listed in** Table 4, we make a long-term investment strategy to reduce the portfolio risk and it is illustrated as follows.

Fourthly, they can select coffee to achieve risk diversification for portfolios in the agricultural market because coffee is the least active asset in the correlation network for two subperiods as shown in Figure 5 (a) and Figure 5 (b). Moreover, as reported in the data in Table 4, we find that, in the pre-pandemic period, the cr-cf (0.0123), sb-cf (0.0132), and so-cf (0.0022) pair of data respectively have the smallest covariance value within the six corn-based, six soybean-based, and six soybean oil-based pairs of data. This indicates that we can make the following long-term investment strategy to reduce the risk of portfolio in the agricultural market. During the pre-pandemic period, if a portfolio includes corn, soybean, or soybean oil, then the investors can add coffee to reduce the risk for this portfolio because the value of the portfolio’s risk is proportion to the value of covariance. A similar long-term investment strategy also is made in the post-pandemic period.

Note: 1. The **policy implications** proposed in the previous version of the manuscript are based on the following results listed in Table 4. 2. The variance of a bi-asset portfolio is expressed as the following equation. As shown in this equation, the value of the portfolio’s risk measured by the portfolio’s variance is proportion to the value of covariance.

$$h_{P,t}=w_{1}^{2}{\cdot h}_{11,t}+w_{2}^{2}{\cdot h}_{22,t}+2w_{1}{\cdot w}_{2}{\cdot h}_{12,t}$$

where $w_{1}$ and $w_{2}$ are the weights of the first and second component assets of a bi-asset portfolio, respectively. $h_{11,t}$ and $h_{22,t}$ are the variances of the first and second component assets of a bi-asset portfolio at time t, respectively. $h_{12,t}$ denotes the covariance between the two abovementioned assets’ returns at time t.

**Table A1.** The summary results of covariance for the 21 pairs of assets during the pre-pandemic period

| Panel A. Wheat-based pair of data | | | | | | |
| --- | --- | --- | --- | --- | --- | --- |
|  | wh-cr | wh-oa | wh-sb | wh-so | wh-cf | wh-su |
| $\omega_{12}^{B}$ | 0.0552  (0.016)^c^ | 0.0345  (0.014)^b^ | 0.0196  (0.003)^c^ | 0.0745  (0.056) | **0.0112**  (0.031) | 0.0031  (0.000)^c^ |
| Panel B. Corn-based pair of data | | | | | | |
|  | cr-oa | cr-sb | cr-so | cr-cf | cr-su | wh-cr |
| $\omega_{12}^{B}$ | 0.0503  (0.014)^c^ | 0.0228  (0.006)^c^ | 0.0161  (0.007)^b^ | 0.0123  (0.009) | 0.3878  (0.075)^c^ | 0.0552  (0.016)^c^ |
| Panel C. Oat-based pair of data | | | | | | |
|  | oa-sb | oa-so | oa-cf | oa-su | wh-oa | cr-oa |
| $\omega_{12}^{B}$ | 0.0347  (0.011)^c^ | 0.0120  (0.007) | 0.6393  (0.144)^c^ | 0.1157  (0.075) | 0.0345  (0.014)^b^ | 0.0503  (0.014)^c^ |
| Panel D. Soybean-based pair of data | | | | | | |
|  | sb-so | sb-cf | sb-su | wh-sb | cr-sb | oa-sb |
| $\omega_{12}^{B}$ | 0.0158  (0.001)^c^ | **0.0132**  (0.009) | 0.3081  (0.080)^c^ | 0.0196  (0.003)^c^ | 0.0228  (0.006)^c^ | 0.0347  (0.011)^c^ |
| Panel E. Soybean oil-based pair of data | | | | | | |
|  | so-cf | so-su | wh-so | cr-so | oa-so | sb-so |
| $\omega_{12}^{B}$ | **0.0022**  (0.001) | 0.0279  (0.013)^b^ | 0.0745  (0.056) | 0.0161  (0.007)^b^ | 0.0120  (0.007) | 0.0158  (0.001)^c^ |
| Panel F. Coffee-based pair of data | | | | | | |
|  | cf-su | wh-cf | cr-cf | oa-cf | sb-cf | so-cf |
| $\omega_{12}^{B}$ | **0.0547**  (0.017)^c^ | **0.0112**  (0.031) | 0.0123  (0.009) | 0.6393  (0.144)^c^ | **0.0132**  (0.009) | **0.0022**  (0.001) |
| Panel G. Sugar-based pair of data | | | | | | |
|  | wh-su | cr-su | oa-su | sb-su | so-su | cf-su |
| $\omega_{12}^{B}$ | 0.0031  (0.000)^c^ | 0.3878  (0.075)^c^ | 0.1157  (0.075) | 0.3081  (0.080)^c^ | 0.0279  (0.013)^b^ | **0.0547**  (0.017)^c^ |

Note: The number marked in red font underneath a specific pair of data in each panel denotes that this specific pair of data has the smallest covariance value within all pairs of data in the same panel.
