# Supplementary material for: The influence of the COVID-19 pandemic on the short- and long-term interactions in the agricultural market: Evidence from a connectedness network approach
Source: PLoS One. 2023 Dec 6;18(12):e0295294. doi: 10.1371/journal.pone.0295294 (PMC10699634; doi:10.1371/journal.pone.0295294)
Supplement: Attachment — Submitted filename: Response reviewer comment.docx [file pone.0295294.s003.docx]

**Journal Requirements:**
Please review your **reference list** to ensure that it is complete and correct. If you have cited papers that have been retracted, please include the rationale for doing so in the manuscript text, or remove these references and replace them with relevant current references. Any changes to the reference list should be mentioned in the rebuttal letter that accompanies your revised manuscript. If you need to cite a retracted article, indicate the article’s retracted status in the References list and also include a citation and full reference for the retraction notice.

**Response**: Thanks for the editor’s suggestions. We have checked the reference list of this revised version of the manuscript. It is complete and correct. Moreover, we mention any changes to the reference list in the rebuttal letter that accompanies our revised manuscript.

**Reviewers' comments:
Reviewer #1**: Cite the following paper:
1.Bao, Z., & Huang, D. (2022). Reform scientific elections to improve gender equality. Nature Human Behaviour, 6(4), 478–479.

2.Bao, Z., & Huang, D. (2023). Gender-specific favoritism in science. Journal of Economic Behavior & Organization. Article in press.

3.Chen, M., Li, N., Zheng, L., Huang, D., & Wu, B. (2022). Dynamic correlation of market connectivity, risk spillover and abnormal volatility in stock price. Physica A: Statistical Mechanics and Its Applications, 587, 126506.

4.Chen, M., Wang, Y., Wu, B., & Huang, D. (2021). Dynamic analyses of contagion risk and module evolution on the SSE a-shares market based on minimum information entropy. Entropy, 23(4), 434.

5.Li, N., Chen, M., Gao, H., Huang, D., & Yang, X. (2023). Impact of lockdown and government subsidies on rural households at early COVID-19 pandemic in China. China Agricultural Economic Review, 15(1), 109–133.

6.Li, N., Chen, M., & Huang, D. (2022). How Do Logistics Disruptions Affect Rural Households? Evidence from COVID-19 in China. Sustainability, 15(1), 465.

7.Wu, B., Huang, D., & Chen, M. (2023). Estimating contagion mechanism in global equity market with time-zone effect. Financial Management, 52, 543–572.

**Response**: Thanks for the editor’s suggestions. In this version of the manuscript, we review the above seven articles as follows.

1.Bao, Z., & Huang, D. (2022). Reform scientific elections to improve gender equality. Nature Human Behaviour, 6(4), 478–479. <https://doi.org/10.1038/s41562-022-01322-w>

Bao and Huang (2022) used the case of the election of Chinese Academy of Sciences (CAS) and Chinese Academy of Engineering (CAE) members to illustrate the gender imparity in scientific elections. They first introduce the recruiting process and then show evidence of gender disparity from data on the election processes between 2001 and 2013. Finally, they proposed three possible reforms to mitigate gender imparity in scientific elections.

***Note****:* The article of Bao and Huang (2022) can be regarded as the previous research of Bao and Huang (2023). Moreover, the issue explored by Bao and Huang (2022) is about the election of Chinese Academy of Sciences (CAS) and Chinese Academy of Engineering (CAE) members and is not related to the topic of this revised manuscript, the **connectedness network in the agricultural market.** Hence, we don’t know how to cite it.

2.Bao, Z., & Huang, D. (2023). Gender-specific favoritism in science. Journal of Economic Behavior & Organization. Article in press. <https://doi.org/10.1016/j.jebo.2023.07.011>

Bao and Huang (2023) used recruitment data or all candidates’ information from the official websites of the Chinese Academy of Sciences (CAS) and Chinese Academy of Engineering (CAE) from 2001 to 2013 as the data and applied the panel data regression model to estimate the gender gap in the election outcomes and further study the presence of such gender-specific favoritism. They found that favoritism from some social connections, such as sharing a hometown, college, or employer, between recruiters and candidates, can benefit men more than women. These results indicate the existence of gender imparity after controlling for social connections

***Note:*** The issue explored by Bao and Huang (2023) is about the election of Chinese Academy of Sciences (CAS) and Chinese Academy of Engineering (CAE) members and is not related to the topic of this revised manuscript, the **connectedness network in the agricultural market.** Hence, we don’t know how to cite it.

3.Chen, M., Li, N., Zheng, L., Huang, D., & Wu, B. (2022). Dynamic correlation of market connectivity, risk spillover and abnormal volatility in stock price. Physica A: Statistical Mechanics and Its Applications, 587, 126506. <https://doi.org/10.1016/j.physa.2021.126506>

Chen et al. (2022) used the SSE A-shares market’s weekly data and apply Pearson correlations of returns, the maximum strongly connected subgraph, and 3σ principle to iteratively determine the threshold value for building a dynamic correlation network of SSE A-shares. Then they compared the differences in network structures between abnormal volatility and calm periods with sharp rises and falls in the market, and identified the difference between abnormal volatility and calm periods from different dimensions. They found that the SSE A-shares network shows a more significant aggregation effect and **stronger connectivity in turbulent periods than tranquil ones**, and the connectivity and aggregation of the SSE A-shares network in 2015 are stronger than those in 2008.

***Note***: The issue explored by Chen et al. (2022) is to compare the differences in network structures of the SSE A-shares market between abnormal volatility and calm periods. The above issue is similar to the topic of this manuscript, comparing the differences in the **connectedness network of the agricultural market** between pre-pandemic and post-pandemic periods. Moreover, Chen et al. (2022) and this manuscript have the same finding, a stronger connectivity in turbulent periods than in tranquil ones. Because the turbulent and tranquil periods in Chen et al. (2022) are almost equivalent to the post-pandemic and pre-pandemic periods in this revised manuscript, respectively. Hence, we cite it in **the last paragraph** of **Section 4.1** ‘The Influence of the COVID-19 Pandemic on the Return Connectedness’ in this revised manuscript, and it is illustrated as follows.

This implies that this pandemic enhances the intensity of return spillover. This result is in harmony with that found by Kang et al. (2017), Mensi et al. (2021), Nekhili et al. (2021), Dai and Zhu (2022), Farid et al. (2022), and Chen et al. (2022) but is similar to that found by Chen et al. (2021). Because most of them found that these spillovers are always intensified in volatile periods caused by economic and political events such as the GFC in Kang et al. (2017), Nekhili et al. (2021), and Chen et al. (2022) as well as the COVID-19 pandemic in Nekhili et al. (2021) and Farid et al. (2022).

4.Chen, M., Wang, Y., Wu, B., & Huang, D. (2021). Dynamic analyses of contagion risk and module evolution on the SSE a-shares market based on minimum information entropy. Entropy, 23(4), 434. <https://doi.org/10.3390/e23040434>

Chen et al. (2021) used the SSE A-shares market’s daily data and employed the Least Absolute Shrinkage and Selection Operator (LASSO) method to construct the stock network as well as the Map Equation method to conduct a dynamic analysis of financial contagion patterns in the SSE A-shares market. They found that the connected effect is more significant in bear markets than bull markets and gives rise to abnormal volatilities in the stock market. Moreover, medium- and small-cap stocks with poor financial conditions are more likely to become risk sources, especially in bear markets.

***Note:*** The issue explored by Chen et al. (2021) is to compare the differences in network structures of the SSE A-shares market between bear markets and bull markets. The above issue is slightly similar to the topic of this manuscript, comparing the differences in the c**onnectedness network of the agricultural market** between the pre-pandemic and post-pandemic periods. Moreover, Chen et al. (2021) and this manuscript have different findings. The reasons are that in Chen et al. (2021), the connected effect is more significant in bear markets than in bull markets whereas in this revised manuscript, the connected effect is more significant in the post-pandemic period than in the pre-pandemic period. Notably, the bear market and bull market in Chen et al. (2021) are almost not equivalent to the post-pandemic and pre-pandemic periods in this revised manuscript, respectively. However, we try to cite it in **the last paragraph** of **Section 4.1** ‘The Influence of the COVID-19 Pandemic on the Return Connectedness’ in this revised manuscript, and it is illustrated as follows.

This implies that this pandemic enhances the intensity of return spillover. This result is in harmony with that found by Kang et al. (2017), Mensi et al. (2021), Nekhili et al. (2021), Dai and Zhu (2022), Farid et al. (2022), and Chen et al. (2022) but is similar to that found by Chen et al. (2021). Because most of them found that these spillovers are always intensified in volatile periods caused by economic and political events such as the GFC in Kang et al. (2017), Nekhili et al. (2021), and Chen et al. (2022) as well as the COVID-19 pandemic in Nekhili et al. (2021) and Farid et al. (2022).

5.Li, N., Chen, M., Gao, H., Huang, D., & Yang, X. (2023). Impact of lockdown and government subsidies on rural households at early COVID-19 pandemic in China. China Agricultural Economic Review, 15(1), 109–133. [https://doi.org/10.1108/CAER-12-2021-0239](https://doi.org/10.1016/j.eneco.2020.104741)

Li et al. (2023) used the data from a national telephone survey of rural households in the early stages of the COVID-19 pandemic, then applied the ordered probit model and linear regression model to examine the impact of lockdown and government subsidies on rural households in early COVID-19 pandemic in China. They found that logistics disruption due to the lockdown negatively affected rural households. Moreover, breeding, transport, and sales suffer more from logistics than cultivation. The reason is that the traffic restriction between and within cities is directly reflected in the serious hindrance of logistics, which leads to production problems such as price rise, production means shortage, sales reduction, and, finally, loss of rural households.

***Note:*** The issue explored by Li et al. (2023) is to discuss the influence of rural households caused by logistics disruption due to the lockdown, a restrictive policy attributed to the COVID-19 pandemic. Hence, we cite it in the **first paragraph** of the **Introduction** in this revised manuscript and it is illustrated as follows.

Moreover, the above policies also made agricultural products from planting to transportation run not swimmingly (Mahajan and Tomar, 2020; Ruan et al. 2021). For instance, the lockdown measures resulted in logistics disruption because of the interruption to transportation, and this disruption further caused a series of problems in agricultural production such as price rise, production means shortage, sales reduction, and, finally, rural households’ losses (Li et al., 2022; Li et al., 2023)

6.Li, N., Chen, M., & Huang, D. (2022). How Do Logistics Disruptions Affect Rural Households? Evidence from COVID-19 in China. Sustainability, 15(1), 465. <https://doi.org/10.3390/su15010465>

Li et al. (2022) used a comprehensive survey of rural households during the early stage of the COVID-19 pandemic in China, then applied a linear probability model or a linear regression with OLS estimation to examine the impact of logistics disruption on rural household losses and further examine the mechanisms by which logistics disruptions affect rural households. They found that logistics disruptions due to the lockdown have resulted in severe economic losses for rural households via an increase in output prices and sales reduction in sales channels, and an increase in input prices, material shortages, capital shortages, and employee shortages in production channels.

***Note:*** The issue explored by Li et al. (2022) is to discuss the influence of rural households caused by logistics disruption due to the lockdown, a restrictive policy attributed to the COVID-19 pandemic. Moreover, the issue explored by Li et al. (2022) is almost the same as that investigated by Li et al. (2023). Hence, we cite it in the **first paragraph** of the **Introduction** in this revised manuscript and it is illustrated as follows.

Moreover, the above policies also made agricultural products from planting to transportation run not swimmingly (Mahajan and Tomar, 2020; Ruan et al. 2021). For instance, the lockdown measures resulted in logistics disruption because of the interruption to transportation, and this disruption further caused a series of problems in agricultural production such as price rise, production means shortage, sales reduction, and, finally, rural households’ losses (Li et al., 2022; Li et al., 2023).

7.Wu, B., Huang, D., & Chen, M. (2023). Estimating contagion mechanism in global equity market with time-zone effect. Financial Management, 52, 543–572. <https://doi.org/10.1111/fima.12430>

Wu et al. (2023) used daily data from 36 national stock indexes and employed a time-zone vector autoregressive (VAR) model to investigate how stock markets in different countries interact with others for the subprime, European debt, and COVID-19 crises. They found the existence of the transmission mechanism in all periods but during the crises, abnormal structural changes in the transmission mechanism. In addition, signed networks provide more information on global equity markets and better identify critical contagion patterns than unsigned networks.

***Note:*** The finding in Wu et al. (2023) can link to that in this manuscript. Hence, we cite it in the **fourth paragraph** of the **Section 5 ‘Conclusion and further discussion’** in this revised manuscript and it is illustrated as follows.

In summary, in the agriculture market, the COVID-19 pandemic impacts the volatility and return connectedness of commodities but not the correlation connectedness of commodities. Thus, the COVID-19 pandemic, a short-term extreme event, can impact short-term interactions like volatility and return spillovers but not one long-term interaction like correlation. We can presume that the COVID-19 pandemic made a structural change in the volatility and return connectedness of commodities. This result can be supported by Wu et al. (2023) and Zhou et al. (2022). For instance, Wu et al. (2023) found abnormal structural changes in the transmission mechanism within 36 national stock indexes during the COVID-19 crisis.

**To sum up, I add the following five literature suggested by reviewer in the reference.**

[34] Chen M, Li N, Zheng L, Huang D, Wu B. Dynamic correlation of market connectivity, risk spillover and abnormal volatility in stock price. Physica A: Statistical Mechanics and Its Applications. 2022; 587: 126506. <https://doi.org/10.1016/j.physa.2021.126506>

[35] Chen M, Wang Y, Wu B, Huang D. Dynamic analyses of contagion risk and module evolution on the SSE a-shares market based on minimum information entropy. Entropy. 2021; 23(4): 434. <https://doi.org/10.3390/e23040434>

[6] Li N, Chen M, Gao H, Huang D, Yang X. Impact of lockdown and government subsidies on rural households at early COVID-19 pandemic in China. China Agricultural Economic Review. 2023; 15(1): 109–133. [https://doi.org/10.1108/CAER-12-2021-0239](https://doi.org/10.1016/j.eneco.2020.104741)

[7] Li N., Chen M, Huang D. How do logistics disruptions affect rural households? Evidence from COVID-19 in China. Sustainability. 2022; 15(1): 465. <https://doi.org/10.3390/su15010465>

[36] Wu B, Huang D, Chen M. Estimating contagion mechanism in global equity market with time-zone effect. Financial Management. 2023; 52: 543–572. <https://doi.org/10.1111/fima.12430>

**Reviewer #2:** The authors are suggested to cite the following papers:
1.Wu, B., Huang, D., & Chen, M. (2023). Estimating contagion mechanism in global equity market with time-zone effect. Financial Management, 52, 543–572.

2.Zhang, Y., Huang, D., Xiang, Z., Yang, Y., & Wang, X. (2023). Expressed Sentiment on Social Media During the COVID-19 Pandemic: Evidence from the Lockdown in Shanghai. Available at SSRN 4486863.

3.Zhou, Y., Huang, D., Chen, M., Wang, Y., & Yang, X. (2022). How Did Small Business Respond to Unexpected Shocks? Evidence from a Natural Experiment in China.

**Response**: Thanks for the editor’s suggestions. In this version of the manuscript, we review the above three articles as follows.

1.Wu, B., Huang, D., & Chen, M. (2023). Estimating contagion mechanism in global equity market with time-zone effect. Financial Management, 52, 543–572. <https://doi.org/10.1111/fima.12430>

Wu et al. (2023) used daily data from 36 national stock indexes and employed a time-zone vector autoregressive (VAR) model to investigate how stock markets in different countries interact with others for the subprime, European debt, and COVID-19 crises. They found the existence of the transmission mechanism in all periods but during the crises, abnormal structural changes in the transmission mechanism. In addition, signed networks provide more information on global equity markets and better identify critical contagion patterns than unsigned networks.

***Note:*** The finding in Wu et al. (2023) can link to that in this manuscript. Hence, we cite it in the **fourth paragraph** of the **Section 5 ‘Conclusion and further discussion’** in this revised manuscript and it is illustrated as follows.

In summary, in the agriculture market, the COVID-19 pandemic impacts the volatility and return connectedness of commodities but not the correlation connectedness of commodities. Thus, the COVID-19 pandemic, a short-term extreme event, can impact short-term interactions like volatility and return spillovers but not one long-term interaction like correlation. We can presume that the COVID-19 pandemic made a structural change in the volatility and return connectedness of commodities. This result can be supported by Wu et al. (2023) and Zhou et al. (2022). For instance, Wu et al. (2023) found abnormal structural changes in the transmission mechanism within 36 national stock indexes during the COVID-19 crisis.

2.Zhang, Y., Huang, D., Xiang, Z., Yang, Y., & Wang, X. (2023). Expressed Sentiment on Social Media During the COVID-19 Pandemic: Evidence from the Lockdown in Shanghai. Available at SSRN 4486863. <https://papers.ssrn.com/sol3/papers.cfm?abstract_id=4486863>

Zhang et al. (2023) used a dataset of about 7 million geotagged microblog tweets from the users on the microblog site in China around the time of the COVID-19 lockdown in Shanghai, then applied a fixed effect panel regression approach to examine the impacts of lockdown on residents’ expressed sentiment. They found that the sentiment index for Shanghai decreased significantly, while the sentiment index for non-Shanghai increased. This indicates that there is a significant divergence in sentiment between Shanghai and non-Shanghai residents after the lockdown was implemented. The reason is that lockdowns can generally be effective at reducing the spread of COVID-19.

***Note:*** The issue explored by Zhang et al. (2023) is related to a series of restrictive policies that the governments in many countries enacted to reduce the spread of the virus in the early COVID-19 pandemic in China. Hence, we cite it in the **first paragraph** of the **Section 1 Introduction** in this revised manuscript and it is illustrated as follows.

Everybody knows that food is a crucial article to survive for human beings. However, climate change such as sudden floods or droughts has made agricultural commodities like grains be planted not smoothly in recent years. Moreover, in 2020, the farmers and workers were infected by the coronavirus disease 2019 (COVID-19) pandemic. To reduce the spread of the virus, the governments in many countries enacted a series of restrictive policies such as the social distancing policy and lockdown measures because social distancing lowered the average daily infection cases by 12% in the United States (Huang, 2020; Zhang et al., 2023).

3.Zhou, Y., Huang, D., Chen, M., Wang, Y., & Yang, X. (2022). How Did Small Business Respond to Unexpected Shocks? Evidence from a Natural Experiment in China. <https://papers.ssrn.com/sol3/papers.cfm?abstract_id=4044677>

Zhou et al. (2022) used the quarterly survey data on a panel of representative small business managers with the People’s Bank of China and employed an ordered probit panel regression model to investigate how small businesses respond to unexpected shocks by studying **the change in managerial beliefs** and their impacts on firm-level outcomes during the COVID-19 pandemic. They found that managerial beliefs are consistent with firms’ performance before the pandemic; managers tend to be optimistic and underreact during the peak while becoming pessimistic and overreacting after the peak. After the pandemic is under control, the managerial biases return to levels before the pandemic.

***Note:*** The issue explored by Zhou et al. (2022) is to discuss how small businesses respond to unexpected shocks by studying **the change in managerial beliefs** and their impacts on firm-level outcomes during the COVID-19 pandemic. The above issue is different with the topic of this manuscript, comparing the differences in the c**onnectedness network of the agricultural market** between the pre-pandemic and post-pandemic periods. However, Zhou et al. (2022) and this manuscript have similar results. That is, the relationship between managerial beliefs and firms’ performance in Zhou et al. (2022) and the c**onnectedness network of the agricultural market in this manuscript are different with the pre-COVID-19 and post-COVID-19 periods. Hence,** we try to cite it in **the last paragraph** of **Section 4.1** ‘The Influence of the COVID-19 Pandemic on the Return Connectedness’ in this revised manuscript, and it is illustrated as follows.

In summary, in the agriculture market, the COVID-19 pandemic impacts the volatility and return connectedness of commodities but not the correlation connectedness of commodities. Thus, the COVID-19 pandemic, a short-term extreme event, can impact short-term interactions like volatility and return spillovers but not one long-term interaction like correlation. We can presume that the COVID-19 pandemic made a structural change in the volatility and return connectedness of commodities. This result can be supported by Wu et al. (2023) and Zhou et al. (2022). For instance, Wu et al. (2023) found abnormal structural changes in the transmission mechanism within 36 national stock indexes during the COVID-19 crisis.

**To sum up, I add the following three literature suggested by reviewer in the reference.**

[36] Wu B, Huang D, Chen M. Estimating contagion mechanism in global equity market with time-zone effect. Financial Management. 2023; 52: 543–572. <https://doi.org/10.1111/fima.12430>

[2] Zhang Y, Huang D, Xiang Z, Yang Y, Wang X. Expressed sentiment on social media during the COVID-19 pandemic: Evidence from the lockdown in Shanghai. 2023. Available at SSRN 4486863. <https://papers.ssrn.com/sol3/papers.cfm?abstract_id=4486863>

[37] Zhou Y, Huang D, Chen M, Wang Y, Yang X. How did small business respond to unexpected shocks? Evidence from a natural experiment in China. 2022. <https://papers.ssrn.com/sol3/papers.cfm?abstract_id=4044677>
